# Transgenic mice applications in the study of endometriosis pathogenesis

**DOI:** 10.3389/fcell.2024.1376414

**Published:** 2024-06-12

**Authors:** Yali Zhao, Yao Wang, Pinlang Gu, Lingjin Tuo, Leilei Wang, Shi-Wen Jiang

**Affiliations:** ^1^ Center of Prenatal Diagnosis, Lianyungang Maternal and Child Health Hospital, Lianyungang, Jiangsu, China; ^2^ Department of Gynecology, Lianyungang Maternal and Child Health Hospital, Lianyungang, Jiangsu, China; ^3^ Lianyungang Research Institute for Women’s and Children’s Health, Lianyungang Maternal and Child Health Hospital, Lianyungang, Jiangsu, China

**Keywords:** endometriosis, transgenic endometriosis model, uterine tissue transplantation, angiogenesis, hormonal regulation, inflammation

## Abstract

Endometriosis (EM), characterized by ectopic growth of endometrial tissues and recurrent pelvic pain, is a common disease with severe negative impacts on the life quality of patients. Conventional uterine tissue transplantation-based models have been broadly used to investigate the pathogenic mechanism(s) of EM. Transgenic mice with whole body or uterine/pelvic tissue-specific labelling by the expression of GFP, β-gal or other light-emitting or chromogenic markers enable investigators to analyze the contribution to endometriotic lesions by the donor or recipient side after uterine tissue transplantation. Moreover, when coupled to uterine tissue transplantation, transgenic mice with a specific EM-related gene knocked out or overexpressed make it possible to determine the gene’s *in vivo* role(s) for EM pathogenesis. Furthermore, observations on the rise of *de novo* endometriotic lesions as well as structural/functional changes in the eutopic endometrium or pelvic tissues after gene manipulation will directly relate the cognate gene to the onset of EM. A major advantage of transgenic EM models is their efficiency for analyzing gene interactions with hormonal, dietetic and/or environmental factors. This review summarizes the features/sources/backgrounds of transgenic mice and their applications to EM studies concerning hormonal regulation, angiogenesis and inflammation. Findings from these studies, the advantages/disadvantages of transgenic EM models, and future expectations are also discussed.

## 1 Introduction

Endometriosis (EM) refers to ectopic establishment and growth of endometrioid tissues outside the uterus, including but not limited to the peritoneum, peritoneal mesentery, surface of ovary, and fallopian tube. Rare EM cases with deep involvements of para-aortic or pericolic lymph nodes resembling a malignancy were also reported (Cacciato Insilla et al., 2014; Li et al., 2022). Affecting approximately 5%–10% women of reproductive age, EM represents one of the most common chronic gynecologic diseases (Shafrir et al., 2018). Although periodic low abdomen pain is the characteristic complaint, fever, syncope, frequent urination and urinary incontinence can occur in EM patients. The recurrent pain severely affects the life quality and casts negative psychological impacts, to cause anxiety and depression in many patients (Chen et al., 2016). It was reported that approximately 25%–50% of female infertility patients suffer EM, which may reflect the hormonal dysregulation in both disorders (Meuleman et al., 2009; ASRM, 2012; Macer and Taylor, 2012). Endometriotic lesions often preserve major histological features of eutopic endometrium including the presence of glandular and stromal cells, and responses to estrogen and progesterone. It is noteworthy that endometriotic tissues often exhibit aggressive behaviors such as invasive implantation and active angiogenesis. It was reported that EM patients have a tripled risk for ovarian cancer, and the rising of clear cell and endometrioid ovarian cancers might be related to EM (van Leeuwen et al., 2011; Anglesio and Yong, 2017). 

According to the 2022 version of ESHRE Endometriosis guidelines (Becker et al., 2022), EM is diagnosed with positive clinical findings, diagnostic laparoscopy, ultrasound and MRI, empirical treatment, and measurement of biomarkers in endometrial tissue, blood, menstrual or uterine fluids. Currently there is no definitive treatment for EM, and hormonal treatment (combined hormonal contraceptives, progestogens, GnRH agonists or GnRH antagonists) and surgical treatment are recommended options to reduce endometriosis-associated pain. Hormonal dysregulation, retrograde menstruation and genetic background are thought to be the etiologic factors. Extensive epigenetic changes, including aberrant patterns of DNA methylation/histone modification and altered noncoding RNA expression, have been found in endometriotic lesions. In EM patients, the endometrial and endometriotic epithelial cells also harbor multiple cancer driver mutations, which may be associated with the establishment of pelvic endometriosis or ovarian cancer (Bulun et al., 2019). Studies also indicated that multifocal endometriotic lesions associated with cancer are clonal and carry a high mutation burden (Anglesio et al., 2015). KRAS somatic mutations were found to be more frequent in patients with deep infiltrating endometriosis or endometrioma lesions and patients with mixed subtypes, compared to those with superficial endometriosis only (Orr et al., 2023). In spite of these findings, the cellular and molecular mechanisms of EM are far from being understood, which have impeded the development of novel therapies. Animal models, especially the endometrial tissue transplantation-based mouse models are broadly employed for EM studies to fill the gap between *in vitro* studies and clinical investigations.

The occurrence of EM displays a clear trend of familial aggregation, with the offspring of an EM mother having 3–9 fold higher risk of suffering the disease (Kim et al., 2021). The first-degree relatives of an EM probands are 7-times more likely to be diagnosed with EM (Nouri et al., 2010). Among the monozygotic twins, if one sister has EM, the possibility for EM in the other one reaches a high level of 75% (Moen, 1994). These observations indicate strong genetic involvements in EM development, despite the fact that no dominant gene or gene mutation has been identified so far, which is not surprising for a common chronic disease. With the fast advancement of *in vivo* gene manipulation techniques, transgenic mouse models have been extensively applied to EM studies. The combination of transgenic approach with uterine tissue transplantation procedures has been used to determine if a pro- or anti-EM effect by a specific gene product could come from the donor side, the host sides, or both. Moreover, by the addition of interfering reagents, transgenic mouse models can be used to analyze the gene interaction with hormonal or environmental factors during EM pathogenesis.

This review summarizes the establishment of, and findings from, the uterine tissue transplantation-based transgenic EM models as well as *de novo* EM models concerning the hormonal regulation, angiogenesis and inflammation, aspects of the disease. Various applications of these models, their advantages/disadvantages, critical issues and future expectations are also discussed. To conduct a thorough but focused review, relevant references were first searched with keywords such as “endometriosis,” “transgenic,” “endometriotic lesion,” and “animal model.” The abstracts of candidate publications were subsequently examined to determine if the full length paper should be requested. We focused on those with high creativity, applying advanced techniques, and/or generating significant impacts on the understanding of EM pathogenesis. Full lengths of chosen publications were carefully read and extensively discussed among the authors.

## 2 Transgenic EM models used to study hormonal regulation

Estrogen dependence and progesterone resistance is the characteristic features of EM, which constitutes a basis for hormonal therapy. The ER/PR gene knockout mice generated in 1990s were among the first generation of transgenic animals, which have opened the door for *in vivo* studies on the hormonal involvements in EM pathogenesis, often by a uterine tissue transplantation approach. For a convenient description, the term “auto-transplantation” here means the transfer of a mouse’s uterine tissues to the pelvic region of the same mouse, and “hetero-transplantation” refers to the transfer of uterine tissues from 1 mouse (donor) to the pelvic region of another mouse (recipient).

Fang et al. (2004) determined the effects of estrogen and progesterone on EM development using the PR knockout (PRKO) mice (C57BL/6/129Sv background) generated by Lydon et al. (1995). Following removal of ovaries, 3 equal-sized pieces of uterine endometrium were auto-transplanted into the bowel mesenteries, in the following 7 groups with or without treatment of E2 (estradiol, 10 μg/day slow-release pellet) and P (progesterone, 250 μg/day slow-release pellet): 1) PRKO, untreated; 2) PRKO, E2-treated; 3) PRKO, E2-and P-treated; and 3 correspondent wild type (WT) controls: 4) WT, untreated; 5) WT, E2-treated; 6) WT, E2-and P-treated. In addition, a WT P-treated group (group 7) was included to verify the progesterone effect. After 8 weeks of continued post-operational treatment with hormones, mice were sacrificed, and ectopic uterine tissues were dissected and their volumes measured. First, between the 2 untreated groups, the PRKO group produced significantly larger ectopic uterine tissues than WT group did. Second, E2 treatment significantly increased the volumes of ectopic uterine tissues, in both PRKO and WT mice. Third, progesterone treatment decreased the volumes of ectopic uterine tissues in WT mice. Also, in WT mice, simultaneous treatment with E2 and P led to significantly smaller ectopic uterine tissues than E2 treatment alone, and this effect disappeared in the PRKO mice. The results of Western blotting assay using protein extracts from ectopic uterine tissues showed altered PCNA (proliferating cell nuclear antigen) levels consistent with the changes in the volumes of ectopic tissues. The authors concluded that intact PR in ectopic uterine tissues was able to abolish the E2-dependent as well as E2-independent endometriotic growth, and suggested that the ectopic growth of uterine tissues was associated with an increased resistance to progesterone, e.g., a propensity to E2-dependent growth by PR deficiency.

To investigate the specific functions of ERα and ERβ in EM, Burns et al. (2012) used the ERα knockout mice (αERKO) (C57BL/6N background) generated by Hewitt et al. (2010), and ERβ knockout mice (βERKO) (C57BL/6 background) produced by Dupont et al. (2000), to perform hetero-transplantation experiments in 5 groups (donor-recipient): WT-WT as a general control, WT-αERKO and WT-βERKO groups, and αERKO-WT and βERKO-WT groups for reverse transplantation. The endometrial tissues from donor mice were isolated, minced, and transplanted in the pelvic cavity of recipient mice. Recipients were oophorectomized 2 weeks before transplantation, and for each tissue transfer group, the mice were divided into 2 subgroups that were treated with either E2 (2.5 μg/mouse per week) or corn oil vehicle. The formation of ectopic endometriosis-like lesions was examined after 3 weeks of continued treatment. In all groups except αERKO-WT, E2 treatment significantly increased the weights of ectopic lesions. The E2 effect appeared to be smaller in βERKO-WT group than that in WT-WT group. In comparison, E2 treatment increased the number of lesions, especially in the WT-αERKO group. The αERKO-WT group only generated 1 lesion around the transplantation site, and E2 treatment failed to increase the number of lesions. While the vehicle-treated recipients generated small lesions that were limited to the transplantation area, E2-treated recipients produced lesions at the transplantation site as well as within the intestinal mesentery, the area around the base and body of the uterus, spleen, and associated fat pads. Moreover, E2 treatment in all groups except αERKO-WT resulted in endometriosis-like tissues filled with liquid. Tissue sections showed apparent vascularization, increased number of epithelial cells following E2 treatment, whereas in αERKO-WT group only floating, unadhered endometrial debris were observed. Moreover, immunohistochemical results showed that with vehicle treatment, the WT-WT group display strong staining for the proliferative marker Ki67, and even with E2 treatment, both αERKO-WT and βERKO-WT groups displayed only week Ki67 staining, suggesting that the presence of ERα and ERβ signaling in the lesions was needed for their full proliferative capacity. Subsequent real-time PCR assay confirmed that the expression levels of two ERα target genes, lactoferrin and mucin4, was significantly increased upon E2 treatment in all groups except αERKO-WT. Furthermore, with the use of real-time PCR, fast responses (2 h) to E2 by the vascular marker genes Vegfa, Mmp7, and Timp1 were detected in the uteri of WT mice, but not in those of αERKO mice. Overall, these results demonstrated a critical role(s) of ER-α signaling for estrogen-mediated EM pathogenesis.

In the ovary, placenta and adipose tissues, aromatase P450 (P450arom) enzyme converts C19 steroids to estrogens (Simpson et al., 1994). While P450arom mRNA is not detectable in the eutopic endometrium of disease-free women, it is detected in the endometriotic tissues as well as eutopic endometrium of EM patients (Noble et al., 1996), and E2 is a potent stimulator of P450arom expression in cultured stromal cells derived from the eutopic endometrium as well as ovarian endometriomas of women diagnosed for EM (Noble et al., 1997). Takayama et al. (1998) successfully treated one case of severe postmenopausal endometriosis with the aromatase inhibitor anastrozole. Fang et al. (2002) applied mice with targeted disruption of P450arom gene (ArKO, C57BL/6J background) (Fisher et al., 1998) to endometriosis study. In either ArKO mice or their WT littermates, endometrial tissues were auto-transplanted into the peritoneal cavity. Mice were not oophorectomized, but primed with conjugated estrogens (CE, 10 μg/day) for 2 weeks. After tissue transplantation in the two types of mice, the mice were subgrouped for CE treatment (10 μg/day) or saline as control. At 8-week post-operation, without CE treatment, the transplants remained as small scars in ArKO mice, whereas the transplants grew into round lesions in WT mice. Continued CE treatment for 8 weeks led to significantly increased sizes of transplants in both ArKO and WT mice. Moreover, in WT groups, treatment with the P450arom inhibitor letrozole alone or with letrozole plus CE resulted in significantly smaller lesions as compared to CE treatment groups, and the effect was dose-dependent. Thus, both P450arom gene disruption and P450arom inhibitor had negative impacts on endometriotic growth, suggesting a requirement of the intact P450arom function for EM pathogenesis. Since the negative effect appeared to be stronger in ectopic endometriotic tissues than in eutopic uterine tissues, the authors proposed that the former may rely more on P450arom function than the latter.

It was reported that promoter hypermethylation and epigenetic silencing of PR-B and/or other genes may confer progesterone resistance (Al-Sabbagh et al., 2012). Sirtuin-1 (SIRT1) is a histone as well as non-histone protein deacetylase that contributes to epigenetic silencing. Yoo et al. (2017) observed a coordinated activation of the Kirsten rat sarcoma viral oncogene homolog (KRAS) and overexpression of SIRT1 in the eutopic endometrium of women with endometriosis throughout the menstrual cycle. Kim et al. (2022) confirmed the overexpression of SIRT1 in both the epithelium and stroma components of endometrium from endometriotic women. They subsequently cross the *Pgr* ^*cre/+*^ mice (C57BL/6/129Sv background) originally developed by Soyal et al. (2005) with the SIRT1 transgenic mice (*Rosa26* ^*Sirt1*^) (C57BL/6J background) generated by Sasaki et al. (2014), to produce the *Pgr* ^*cre/+*^ *Rosa26* ^*Sirt1/mTmG*^ strain in which the uterine-specific SIRT1 overexpression was achieved. The mice were oophorectomized and primed with E2 (100 ng/mouse) for 3 days before auto-transplantation. Auto-transplantation in *Pgr* ^*cre/+*^ *Rosa26* ^*Sirt1/mTmG*^ mice resulted in an increased number of endometriotic lesions as compared to *Pgr* ^*cre/+*^ *Rosa26* ^*mTmG/+*^ mice without SIRT1 overexpression. Consistently, oral administration of SIRT1 agonist SRT17201 (1 mg/kg) for 1 month in the *Pgr* ^*cre/+*^ *Rosa26* ^*mTmG/+*^ mice without SIRT1 overexpression led to a significantly increased number of endometriotic lesions. On the contrary, treatment of the SIRT1 overexpression model with the SIRT1 inhibitor EX-527 for 2 weeks had a negative impact on the ectopic growth of uterine tissues. Subsequently, the investigators showed that SIRT1 overexpression resulted in a nonreceptive and progesterone resistant status of endometrium in which the E2-responsive genes were upregulated and progesterone-responsive genes were downregulated. Moreover, the investigators demonstrated a physical interaction of Sirt1 protein with PR-A, but not PR-B, by immunoprecipitation assay. Since immunohistochemistry showed an unchanged PR expression level in the uteri of mice with SIRT1 overexpression, the authors speculated that aberrant SIRT1 expression may confer progesterone resistance through SIRT1-PR-A protein interaction. While the study reveals a promoting role of SIRT1 for EM pathogenesis, more detailed studies are required to clarify the underlying molecular and cellular mechanism.

Prion (PrP^C^), the product of PRNP gene, is a glycosylphosphatidylinositol (GPI)-anchored cell membrane protein. In the mouse uterus, its expression is activated by estrogen, but hindered by progesterone (Ding et al., 2018). In a recent study, Peng et al. (2022) perfomed a transcriptome analysis on human endometriotic lesions and observed a simultaneous upregulation of PrP^C^ with estrogen biosynthesis enzymes (stAR, CYP11A1, CYP19A1 and HSD3B2). In primary cultures of stromal cells isolated from normal human endometrium or ovary endometrial cystic wall, estrogen treatment enhanced the cell survival, PrP^C^ expression, cholesterol accumulation and estrogen biosynthesis. Transcriptomic comparison between stromal cell cultures with and without PrP^C^ overexpression/knockdown suggested that PrP^C^ might contribute to cholesterol metabolism and estrogen biosynthesis through negative modulation of PPARα (Peroxisome proliferator-activated receptor-alpha). The investigators subsequently performed hetero-transplantation of uterine tissues from the following 3 strains of donor transgenic mice with differential PRNP expression status to the peritoneal cavity of WT mice (C57BL/6J background): PRNP whole-gene knockout (*Prnp* ^*−/−*^, KO119, C57BL/6J background), PRNP partial deletion (KO120, C57BL/6J background), and PRNP overexpression (Tg20, C57BL/S129 background), all constructed by the BRL Medicine Company. As a control, uterine tissues from WT mice were also transplanted. Donor mice were primed at day 1 and day 4 with E2 (3 μg/mouse). At 2-week post-operation, uterine tissues from KO119 and KO120 produced smaller and Tg20 produced larger, endometriotic tissues, than those from WT mice did, in WT recipients. Although the status of estrogen biosynthesis and its possible regulation by PPARα was not verified in the mouse endometriotic tissues, the results suggested that PrP^C^ had a promoting role for the ectopic growth of endometriotic tissues, and that PPARα pathway could be a potential therapeutic target for the disease.

Steroid receptor co-activators (SRCs) modulate the nuclear receptor-mediated cell functions in a tissue-selective manner, which enables precise responses to hormonal stimulation. A decreased SRC-1 (p160) protein level was observed in the endometrium of EM patients (Suzuki et al., 2010). Han et al. (2012) crossed the *SRC-1* ^*−/−*^ mice (C57BL/6/129Sv background) (Xu et al., 1998) with whole body-GFP (green fluorescent protein) expressing mice (C57BL/6 background) (Jackson Laboratory), to obtain *SRC-1* ^*−/−*^:GFP mice. After oophorectomy and priming with 17-β-estradiol (embedded pellets, 0.36 mg/day release) for 2 days, uterine tissues from donor mice were hetero-transplanted to the mesenteric membranes of recipients, and their growth was determined 21 days later. Uterine tissues from SRC-1 deficient mice grew significantly smaller lesions than those from WT, in WT recipients. Immunohistochemistry detected lower COX-2 and VEGFR levels in the former tissue transfer group than the latter group. Moreover, treatment with a MMP9 inhibitor (MMP2/9 In) decreased the volumes of ectopic lesions formed by auto-transplantation in WT mice. The subsequent auto-transplantation in *Mmp9* ^*−/−*^ mice (C57BL/6J/FVB background, Jackson Laboratory) showed that MMP9 deficiency led to smaller ectopic lesions than those formed in the congenic FVB/NJ mice. Furthermore, auto-transplantation in TNF-α deficient mice resulted in smaller ectopic lesions and a concomitantly decreased MMP9 activity in the lesions, as compared to WT. MMP9 was predicted to cleavage the intact SRC-1 to produce a 70 kDa C-terminal isoform, and *in vitro* experiments in immortalized human endometrial epithelial cells (IHEECs) showed that this isoform was able to prevent the TNF-α-mediated apoptosis and to promote these cells’ epithelial-mesenchymal transition (EMT) as well as invasiveness. The authors concluded that activation of the TNF-α/MMP9/SRC-1 pathway may promote EM pathogenesis. The coordinated changes among TNF-α, MMP9 and SRC-1 in human EM lesions has not been reported, and the role(s) of SRC-1 70 kDa C-terminal isoform remains to be verified.

Recently, Yoo et al. (2022) observed that the mRNA as well as protein levels of the tumor suppressor gene Mig-6 (mitogen-inducible gene 6) is downregulated in the endometrium of infertile women with endometriosis and in baboon endometriosis model. In mouse endometrial tissue transfer model, the uterine tissues from Mig-6 knockout mice grew bigger number and heavier weight of ectopic endometriotic lesions in wild type mice. Mig-6 knockout led to increased Erbb2 (CD340, HER2/Neu proto-oncogene) expression in the endometrium. While the Mig-6 knockout mice were infertile, the fecundity of mig-5 and Erbb2 double knockout mice were normal. The results indicated that ERBB2 overexpression in endometrium with Mig-6 deficiency causes endometrial progesterone resistance and a non-receptive endometrium in endometriosis-related infertility.

## 3 Transgenic mice used to study angiogenesis in EM

Active angiogenesis can be observed in both eutopic uterine tissue and EM lesion, and high expression of angiogenic genes such as vascular endothelial growth factor-A (VEGF-A) and hepatocyte growth factor (HGF) was detected in ectopic endometriotic tissues (Delbandi et al., 2020). In a chicken chorioallantoic membrane model, the formation of endometriosis-like lesions was significantly inhibited by angiostatic reagents, and the effect was accompanied by a decreased vascularization as well as an increased necrosis in the endometriosis-like lesions (Nap et al., 2005).

It has been shown that leptin, a food intake regulator, may contribute to angiogenesis (Sierra-Honigmann et al., 1998; Herrera-Vargas et al., 2021). Most studies, but not all, observed increased leptin levels in the serum and/or peritoneal fluids of EM patients (Matarese et al., 2000; Mahutte et al., 2003; Kalaitzopoulos et al., 2021). Styer et al. (2008) applied the leptin receptor mutant mice (Lepr^db^, C57BL/6J background) (Johnson and Sidman, 1979) to investigate the role(s) of leptin-receptor signaling in EM development. Donor mice were oophorectomized and primed with 17-β-estradiol implants for 5 days before harvest of uterine tissues. First, the endometrial tissues from WT were transplanted to the peritoneal cavity of syngeneic sisters, and the recipient mice were treated with LPrA (leptin peptide receptor antagonist), or LPrASc (scrambled peptide), goat IgG, and DMSO solvent as respective controls, 1 day before transplantation. The formation of EM-like lesions was determined after 7 or 14 days of transplantation and continued treatment. The LPrA treatment group displayed a unique ectopic lesion phenotype characterized by minimal vascularization, extensive fibrosis and sclerosis, with rare presence of endometrial glandular epithelium and stroma. In the control groups, the ectopic lesions were well-vascularized, with significant peritoneal attachment, and typical endometrial glandular and stromal architecture. The inhibitory effect of LPrA was confirmed by transferring the GFP-positive uterine tissues to GFP-negative recipients and subsequent measurement on the fluorescence strength of viable EM-like tissues. There was EM-like lesion formation in both of the host groups (WT and Lepr^db^), but the gross morphology and the histologic appearances and features of these EM-like lesions were different. Critically, while the transfer of endometrial tissues from Lepr^db^ donor mice to WT littermates produced EM-like lesions with typical endometrial morphology and histology, the reverse transfer led to unusual morphology and histology. This result strongly suggested that the leptin-receptor system may play a role(s) for EM pathogenesis by regulating some key events in the recipient side, rather than donor side. Moreover, immunohistochemistry with VEGF antibody indicated that VEGF was expressed two-fold lower in the lesions from LPrA treatment group than lesions from control groups, suggesting that leptin-receptor might exert its effects through affecting the VEGF expression. Importantly, overexpression of an endogenous VEGF receptor antagonist, sFlt-1, in recipient mice through tail vein injection of sFlt-1-expressing adenovirus, led to lesions with minimal vascularity, and minimal phosphohistone H3 (a cell proliferation marker) and VEGF staining. Moreover, treatment of Lepr^db^ host mice with recombinant VEGF resulted in ectopic lesions with significant necrosis, and glandular/stromal breakdown, rather than formation of typical EM-like lesions. Furthermore, pre-treatment with LPrA for 1 day before tissue transfer and continued treatment following tissue transfer led to a significant reduction of micro vessel density in ectopic lesions, as compared to LPrASc treatment, suggesting that the inhibition of leptin signaling could affect the initial vascular recruitment.

*In vitro* studies indicated that activation of PPARγ may inhibit the proliferation of endometriotic cells through suppression of estrogen biosynthesis (Lebovic et al., 2013; Vallée et al., 2021). Resveratrol, an anti-inflammation and anti-oxidation phytoestrogen, displays a potential therapeutic effect for EM in several animal models (Kolahdouz Mohammadi and Arablou, 2017). Interestingly, resveratrol was able to reduce the size of transplanted endometrial tissues, possibly through activation of PPARα (Chen et al., 2021). Pergialiotis et al. (2022) performed auto-transplantations in WT C57BL6 mice and PPARα-knockout mice (C57BL/6/129S4 background, Jackson laboratory), respectively. The ectopic uterine tissues were sutured in the pelvic cavity to ensure their direct contact with the peritoneal surface. At 14-day post-operation, 50% of WT mice had endometriotic crypts, but only 10% of PPARα KO mice developed endometriotic crypts. Histological examination showed that while 80% of WT mice displayed significant neo-vascularization in their endometriotic tissues, only in half of PPARα KO mice the endometriotic tissues had minimal neo-vascularization. The authors concluded that PPARα may contribute to EM pathogenesis, and PPARα may serve as a therapeutic target. It is unclear if PPARα might exert its activity in the ectopic endometrial cells/tissues or pelvic side, or both, in this specific model as well as in a general manner.

Accumulated data support a central role of the Slit-roundabout signaling for cancer-related angiogenesis as well as physiological angiogenesis during normal development. Guo et al. (2013) applied the Slit2 overexpression mice (Slit2 Tg, C57BL/6 background), in which the expression of Slit2 transgene was driven by the CMV promoter (Yang et al., 2010), to study endometriosis. Hetero-transplantation of uterine tissues to the peritoneal cavity of recipients or sham operation without tissue transfer were carried out in the following 6 groups: Slit2 Tg-Slit2 Tg; WT-Slit2 Tg; sham operation in Slit2 Tg; WT-WT; Slit2 Tg-WT; sham operation in WT mice. The formation of ectopic lesions was determined at 4-week post-operation. Endometriotic establishment was observed in all the tissue transfer groups, but not in the two sham operation groups. Importantly, the average size of endometriotic lesions in Slit2 Tg-Slit2 Tg group was 3-times larger than that in WT-WT group, and intermediate sized lesions were found in WT-Slit2 Tg and Slit2 Tg-WT groups. Thus, Slit2 overexpression in either donor tissues or host was able to promote endometriotic growth, with the effect maximized by simultaneous overexpression of Slit2 in both sides. Subsequent immunohistochemistry showed a positive correlation of endometriotic growth with micro vessel density, but not with VEGF immuno-reactivity. While the study demonstrated a Slit2-mediated promotion of endometriotic growth and angiogenesis, the underlying mechanisms, especially the involvements of angiogenic factors, remained to be determined.

FKBP52 (immunophilin cochaperone FK506-binding protein 4) assists PR chaperoning by interacting with the PR-Hsp90 complex to modulate the progesterone/PR-mediated transcriptional regulation of target genes (Barent et al., 1998). FKBP52 expression was found to be decreased in the eutopic endometrium during the receptive window in a baboon EM model induced by inoculation of menstrual endometrium into the peritoneal cavity (Jackson et al., 2007). Hirota et al. (2008) observed a decreased FKBP52 expression in the eutopic endometria as well as ectopic lesions from EM patients, and applied the FKBP52 knockout mice (*Fkbp52* ^*−/−*^, C57BL/6/129SvJ background) that was originally generated by Cheung-Flynn et al. (2005) and later crossed with CD1 Swiss mice by Tranguch et al. (2007), for hetero-transplantation. Endometrial tissues from diestrus donor were transplanted to the pelvic cavity of recipient littermates, in 4 groups: WT-WT; *Fkbp52* ^*−/−*^ — *Fkbp52* ^*−/−*^, WT — *Fkbp52* ^*−/−*^, *Fkbp52* ^*−/−*^ — WT. At 2-week post-operation, the *Fkbp52* ^*−/−*^ — *Fkbp52* ^*−/−*^ group grew endometriotic tissues with the highest numbers and weights among all groups. WT-*Fkbp52* ^*−/−*^ and *Fkbp52* ^*−/−*^WT groups generated endometriotic lesions that were in similar quantity but significantly more than WT-WT group did. Thus, Fkbp52 deficiency in both the donor and recipient sides could promote the formation of endometriotic lesions in this model. Subsequent immunohistochemistry indicated that angiogenic regulators COX-2 and VEGF were more expressed in the endometriotic tissues from *Fkbp52* ^*−/−*^ — *Fkbp52* ^*−/−*^ group than those from WT-WT group, suggesting that the Fkbp52 deficiency-triggered angiogenesis might be involved. To verify this effect, the investigators took advantage of the *Flk1* ^*lacZ+/−*^ transgenic mice (C57BL/6J/Sv129 background) (Shalaby et al., 1995) in which the β-galactosidase (lacZ) expression was driven by the Flk1 promoter that is active in nascent vascular epithelial cells. These mice were crossed with *Fkbp52* ^*−/−*^ or WT mice to produce *Fkbp52* ^*−/−*^ */Flk1* ^*lacZ+/−*^ and *Fkbp52* ^*+/+*^ */Flk1* ^*lacZ+/−*^ mice. Positive Lacz staining was found in the endometriotic tissues from WT-*Fkbp52* ^*+/+*^ */Flk1* ^*lacZ+/−*^ and *Fkbp52* ^*−/−*^ — *Fkbp52* ^*−/−*^ */Flk1* ^*lacZ+/−*^ groups, but not in the two correspondent reverse transfer groups. Consistently, the highest micro vessel density was found in the *Fkbp52* ^*−/−*^ — *Fkbp52* ^*−/−*^ */Flk1* ^*lacZ+/−*^ group. These results indicated that the ectopic uterine tissues recruited blood vessels from the recipient side, at least in this specific model. By these reciprocal transfer experiments, the study demonstrated the Fkbp52 deficiency-mediated promotion of EM pathogenesis and a key role(s) of recipient tissues for angiogenesis.

Ono et al. (2021) used the CD206 DTR mice in which the diphtheria toxin receptor was expressed under the control of CD206 promoter to investigate EM pathogenesis (Kambara et al., 2015). Upon induction with diphtheria toxin, 80% of CD206+ macrophages will be ablated in various organs. After endometrial tissue transfer, the depletion of CD206+ macrophages in CD206 DTR mice significantly decreased the total weight of EM-like lesions, but the number of lesions per mouse was not changed compared to control. A lower proliferation of endometriotic cells and the decrease of angiogenesis were observed in the EM-like tissues after depletion of CD206+ macrophages, suggesting that CD206+ macrophages may promote the formation of EM-like lesions by inducing angiogenesis around the lesions.

## 4 Transgenic mice used to study inflammation/immune cell functions in EM

The inflammatory features of endometriotic tissues are evidenced by extensive infiltration of activated macrophages, neutrophils, natural killer cells, and dendritic cells as well as increased levels of cytokines, chemokines, immunoglobulins and complements (Symons et al., 2018). Also, the peritoneal fluids from EM patients contain elevated levels of pro-inflammatory cells and cytokines/chemokines (Symons et al., 2018). Indeed, local inflammatory stimulation of vagus nerves is thought to be the direct cause of pelvic pain associated with EM (Morotti et al., 2017; Patel et al., 2018; Zheng et al., 2019). Co-localization of high densities of macrophages with nerve fibers was observed in peritoneal endometriotic lesions and eutopic peritoneum of EM patients (Tran et al., 2009).

IL-32 represents a major mediator of inflammation in rheumatoid arthritis, chronic obstructive pulmonary disease, chronic rhinosinusitis, ankylosing spondylitis and inflammatory bowel disease (Shioya et al., 2007; Calabrese et al., 2008; Ciccia et al., 2012; Soyka et al., 2012). Following confirmation on a significantly increased IL-32 level in pelvic fluids of EM patients, Lee et al. (2018) carried out auto-transplantation in the IL-32γ-overexpressing transgenic mice (IL-32γ TG, C57BL/6 background) originally created by Choi et al. (2010). Two weeks after oophorectomy, the mice were primed with embedding of E2 capsules, and the uterine horns were transplanted to the peritoneal wall. At 31-day post-operation endometriotic lesions were isolated and measured. The volume of endometriotic lesions in IL-32γ TG mice was significantly larger than that in WT mice. Immunohistochemistry showed that the levels of proliferation markers Ki-67 and PCNA were higher in the lesions from IL-32γ TG mice than those from WT mice. Moreover, IL-32 treatment of Ishikawa endometrial glandular cells or primary endometrial stromal cells isolated from women diagnosed with leiomyoma resulted in an increased cell viability, proliferation and invasiveness. Thus, elevated IL-32 levels exert pro-EM effects in both transgenic EM mouse model and endometrial cell culture. Further mechanistic studies on the IL-32-mediated activation of endometrial cells would help us to better understand the relationship between pro-inflammatory factors and EM progression.

Eicosapentaenoic acid (EPA, C20:5n-3) and docosahexaenoic acid (DHA, C22:6n-3) are omega-3 polyunsaturated fatty acids (Omega-3 PUFAs) with anti-inflammatory activities (Seki et al., 2010). The Fat-1 gene from *C. elegans* encodes an omega-3 fatty acids desaturase that converts omega-6 PUFAs to omega-3 PUFAs, and most mammalians do not possess this gene. Kang et al. (2004) created transgenic mice (Fat-1, C57BL/6 background) expressing the Fat-1 gene, and showed that these mice had an abundance of Omega-3 PUFAs and a reduction in omega-6 PUFAs in the organs and tissues, in the absence of dietary Omega-3 PUFAs. Tomio et al. (2013) applied these mice to investigate the anti-inflammatory function of endogenous Omega-3 PUFAs. After oophorectomy and priming with E2 (100 μg/kg) in donor and recipient mice, hetero-transplantation of uterine tissues to the peritoneal cavity of recipient littermates were carried out. Two weeks after operation the ectopic endometrotic lesions and peritoneal washes were collected. The Fat-1-Fat-1 transfer group produced much fewer endometriotic lesions than WT-WT group did, and the average weight of lesions from Fat-1-Fat-1 was much lighter than that from WT-WT. Lipidomic analysis showed that the level of 12/15-hydroxyeicosapentaenoic acids (12/15-HEPE), an EPA metabolite, was significantly higher in the lesions from Fat-1-Fat-1 group than those from WT-WT group, but the DHA level was unchanged. To investigate EPA effects, the 12/15-LOX-KO mice (C57BL/6 background, Jackson Laboratory) that are deficient in conversion of EPA to 12/15-HEPE were employed for uterine tissue transfer and EPA administration experiments. Results showed that oral administration of EPA significantly decreased the number of lesions in WT-WT group, but not in 12/15-LOX-KO-12/15-LOX-KO group, suggesting that the conversion of EPA to 12/15-HEPE could be an important step for the inhibitory effect of EPA on EM progression. The anti-inflammatory effect of Omega-3 PUFAs was demonstrated by cDNA microarray results that showed a decreased expression of multiple pro-inflammatory cytokines in the lesions from Fat-1-Fat-1, with the most reduction detected in IL-6. Subsequent isolation of macrophages from peritoneal cavity and real-time PCR measurement of IL-6 mRNA revealed a consistent change. The authors proposed that both endogenous and exogenous EPA-derived PUFAs may protect against the pathogenesis of endometriosis through their anti-inflammatory effects, with the 12/15-LOX-pathway products of EPA serving as key mediators.

Tal et al. (2021) performed tissue transfer by suturing the uteri from female mice homozygous for floxed alleles of C-X-C chemokine receptor type 4 (CXCR4) and co-expressing Cre recombinase under the control of progesterone receptor promoter onto the peritoneum of host mice expressing GFP. Donor uteri with conditional knockout of CXCR4 developed significantly lower number of lesions than controls. while CD3^+^ lymphocytes were largely excluded from the epithelial compartment in control lesions, CD3^+^ lymphocytes infiltrated the Cxcr4-deficient epithelium in the diestrus and proestrus stages. The data suggested that local CXCR4 expression might be required for the proliferation of the epithelial compartment in endometriosis lesions.

## 5 The use of Pten knockout mice for EM studies

PTEN tumor suppressor gene product is a phosphatase that downregulates the PI3K/AKT pathway (Cantley and Neel, 1999). Accumulated data indicated an over-activation of PI3K/AKT pathway in endometriotic stromal cells (Cinar et al., 2009; Yin et al., 2012). Daikoku et al. (2008) crossed the *PR* ^*cre*^ mice (C57BL/6/129Sv background) (Soyal et al., 2005) with the *Pten* ^*f/f*^ mice (C57BL/6J background, Jackson Laboratory) to generate the bigenetic *Pten*-heterologous *PR* ^*cre/+*^ *Pten* ^*f/+*^ mice in which 1 allele of *Pten* is abrogated specifically in PR-expressing cells. The homozygous mice with 2 *Pten* alleles knocked out developed early onset (1 month) endometrial cancers, but 20% *Pten*-heterologous females developed endometrial cancers and all females had atypical endometrial hyperplasia at 10 months of age. Kim et al. (2014) performed immunohistochemistry to demonstrate that the endometrium of *Pten*-heterologous mice had a diminished level of p (Ser473)-AKT. However, after removal of ovaries and treatment with E2 (0.36 mg/day for 60 days), the level of p (Ser473)-AKT elevated, suggesting an AKT activation by E2. Subsequently, auto-transplantation of uterine tissues to the pelvic cavity was carried out 2 weeks after oophorectomy and E2 priming (0.1 μg/day, for 3 days). After 4 weeks of continued treatment with embedded E2 pellets (releasing 0.36 mg/day for 60 days), the *PR* ^*cre/+*^ *Pten* ^*f/+*^ mice produced significantly more endometriotic lesions than *Pten* ^*f/+*^ control mice with 2 intact *Pten* alleles did. Oral gavage with AKT inhibitor MK-2206 (360 mg/kg, once a week) or vehicle for 4 weeks resulted in a significantly reduced number of endometriotic lesions in both *PR* ^*cre/+*^ *Pten* ^*f/+*^ and *Pten* ^*f/+*^ mice compared to vehicle-treated mice. In addition, the difference in the number of endometriotic lesions between *PR* ^*cre/+*^ *Pten* ^*f/+*^ and *Pten* ^*f/+*^ mice disappeared after treatment with AKT inhibitor, indicating the cancelation of *Pten* deficiency-mediated effects by AKT inhibitor. Consistently, immunohistochemistry results showed decreased levels of p-AKT in the ectopic tissues associated with AKT inhibitor treatment. Taken together, the findings strongly indicated a promoting role of AKT activation for EM pathogenesis.

## 6 Transgenic mice with *de novo* EM

All the above discussed models are based on forced tissue transplantation, and the experimental outcomes will more or less, but inevitably, be affected by transplantation procedures, which weakens the convincing power of the results. In contrast, if manipulation of a specific gene led to the *de novo* occurrence of EM, the results would provide solid evidence in support of this gene’s primary role for the disease. So far only one *de novo* EM transgenic model has been reported.

In 2001, Johnson et al. (2001) reported that a strain of mice (*LSL-K-ras* ^G12D/+^, C57BL/6/129/Sv background) engineered to express the oncogenic *K-ras* gene that harbors a glycine to aspartic mutation in the exon 1, had early onset of lung cancers. The tissue-specific expression of oncogenic *K-ras* mutant was controlled by local injection of an adenovirus (AdCre) expressing the Cre enzyme to excise the stop codon preceding the *K-ras* coding sequences. Dinulescu et al. (2005) showed that 8 months after activation of the oncogenic *K-ras* allele in the ovarian surface epithelium (OSE) by injection of AdCre to the bursal cavity, benign epithelial lesions with simple endometrioid glandular morphology reminiscent of the epithelial component of endometriosis occurred in OSE. The lesions were considered endometriosis-like lesions due to their lack of typical endometrial stroma. No endometriosis-like lesion was observed in the *LSL-K-ras* ^G12D/+^ mice without bursal cavity injection of AdCre or in WT mice with AdCre injection. Interestingly, in addition to the lesions in OSE, peritoneal endometriosis with complete glandular and stromal components in the pelvic peritoneum and soft tissues around ovary were observed in 47% of *LSL-K-ras* ^G12D/+^ mice after bursal cavity injection of AdCre. Analysis on the morphological characters and immunohistochemical identification of a variety of uterine epithelial markers revealed a high similarity of the peritoneal lesions to the eutopic uterine tissues, confirming these lesions’ endometriosis nature. Since AdCre injection into the peritoneum did not produce endometriosis, the peritoneal endometriosis observed after bursal cavity injection of AdCre did not appear to originate from metaplastic transformation of pelvic peritoneum.

To further delineate the tissue origin of endometriosis-like lesions on the ovary surface and the peritoneal endometriosis, ovary transplantation was performed. AdCre was injected into the bursal cavity of the *LSL-K-ras* ^G12D/+^ mice, 48 h later the ovaries from these mice were transplanted under the ovarian bursa of BALB/c Rag2^−/−^ mice with immune deficiency. Observation 5.5 months later showed that all the recipient mice developed ovarian endometriosis-like lesions, but not peritoneal endometriosis. The authors proposed a possibility that at least in the *LSL-K-ras* ^G12D/+^ model, the ovarian and peritoneal lesions may have distinct origins, with the ovarian lesions arising from the OSE, with the peritoneal lesions arising from the uterine or tubal origin.

## 7 The use of transgenic mice expressing marker proteins for EM studies

Transgenic mice expressing common markers such as GFP, β-galactosidase (β-gal, encoded by LacZ gene) or luciferase have all been applied to EM studies. GFP, especially EGFP (enhanced GFP) capable of emitting much brighter fluorescent light, can be conveniently detected *in vivo*, or in fixed tissues for gross or microscopic observations upon excitation with blue lights, without a need of substrate or other condition. GFP can also be appended to the amino or carboxyl terminus of a protein without a loss of the protein activity or GFP function. β-gal is an enzyme catalyzing the hydrolysis of X-gal to produce the chromogenic dichloroindigo that is visualized as blue color in dissected tissues as well as tissue sections after staining with X-gal. Luciferase catalyzes the oxidation of luciferin the bioluminescence emitter oxyluciferin in the presence of ATP. Luciferase assay is of low background, and often performed *in vitro* with isolated tissue extracts on an illuminometer. Taking advantages of these markers expressed by transgenic mice, uterine tissue transfer-based EM models can be applied in the following ways.

### 7.1 Transgenic mice with whole body expression of GFP or β-gal

Transgenic mice with whole body expression of GFP or other markers driven by a viral or commonly active promoter can be used as the donors of uterine tissues. After hetero-transplantation, the ectopic cells/tissues can be easily distinguished from the host cells/tissues. Thus, characterization of the ectopic lesions’ establishment, proliferation, and other features becomes more accurate and convenient. In reverse, when such mice are used as recipients, the labeling of recipient cells/tissues makes it possible to determine the contribution to EM lesions by the host entities. For example, Okabe et al. (1997) created transgenic mice in which the GFP expression was driven by the chicken β-actin promoter and cytomegalovirus enhancer. GFP expression was observed in all tissues except erythrocytes and hair. Applying this model and intravital fluorescence microscopy, Feng et al. (2014) was able to show that the presence of uterine luminal epithelium in the donor tissues negatively affected the ectopic growth of endometriosis-like lesions in the dorsal skinfold chamber of GFP-negative recipient mice, possibly by preventing the vascular interconnection with the microvasculature of the surrounding host tissues. Similar methods have been successfully applied to investigate the attachment and invasion (Machado et al., 2014), endometriotic lesion growth and progression (Ferrero et al., 2017), cellular interplay between ectopic tissue and host peritoneum (Wilkosz et al., 2011), and inhibition of endometriotic growth by all-trans-retinoic acid (Wieser et al., 2012). Recently, Wibisono et al. (2022) created a new transgenic mouse with whole body expression of emerald luciferase (ELuc) under the control of the CAG promoter. The mouse showed strong bioluminescence emission, which was used to tracing the EM-like lesions after tissue transfer. The accuracy of ELuc-mediated tracing was high and depended on the dosage of E2 administration. Kim and Bae (2022) used the diet-induced and genetically engineered obese mice and fluorescence-tagged ectopic lesions to demonstrate that in obese recipient mice with leptin deficiency and leptin receptor deficiency the development of EM-like phenotype was suppressed. The authors concluded that leptin and its receptor are critical for endometriosis development.

### 7.2 Transgenic mice expressing a marker gene in a uterine cell- or tissue-specific manner

In this case, the expression of a marker protein is driven by a uterine cell- or tissue-specific promoter. This nifty design makes it possible to characterize a pathologic mechanism paramount to EM, e.g., whether an effect is dominated by the uterine endometrial stromal or epithelial lineages. Shalaby et al. (1995) introduced the lacZ gene into mice and β-gal expression was driven by the Flk1 promoter that is active in vascular epithelial cells. As discussed in detail above, applying these mice to uterine tissue transfer, Hirota et al. (2008) obtained compelling evidence in support of the host origin of vessels in endometriotic lesions, especially the vascular epithelial cells from surrounding pelvic tissues. In another study, Patterson et al. (2013) used the transgenic mice expressing either EYFP (enhanced yellow fluorescent protein) (C57BL/6J/s 129X1/SvJ background, Jackson laboratory) or β-gal (C57BL/6 background) (Arango et al., 2008) to demonstrate that EMT contributed to endometrial regeneration during natural as well as artificial decidualization. Since the expression of EYFP and β-gal markers was driven by the Amhr2 promoter that is only active in the mesenchymal cells, these cells’ transition to epithelial cells and relocation to the regeneration zone of the mesometrial endometrium was accurately traced. The findings suggested that EMT might be implicated in proliferative disorders of endometrium such as EM.

### 7.3 Transgenic mice for *in vivo* characterization of gene regulation

Uterus, as a reproductive organ with dramatic menstrual and gestational changes, its cell-/tissue-specific, and spatial-temporal gene regulations constitute a basis for this organ’s physio-pathological functions. For *in vitro* studies, coding sequences for chromogenic or light-emitting reporters are frequently inserted downstream of a gene’s promoter to investigate the cognate gene’s regulation by the cis-elements and/or transcriptional factors. While *in vivo* reporter assays by transgenic approach has been used for studying other organs/disorders, the method has not been applied to EM study so far.

### 7.4 Transgenic mice expressing marker-fused proteins

GFP and a variety of antigenic or affinity tags/epitopes can be linked to a target protein, to produce fusion proteins for convenient detection. This approach can be used to investigate protein localization, translocation, subcellular structures, and/or protein-protein interactions. Although readily applied to *in vitro* studies in cell cultures, the approach has not been applied to *in vivo* EM study in transgenic mice.

## 8 Conclusion

**FIGURE 1:**
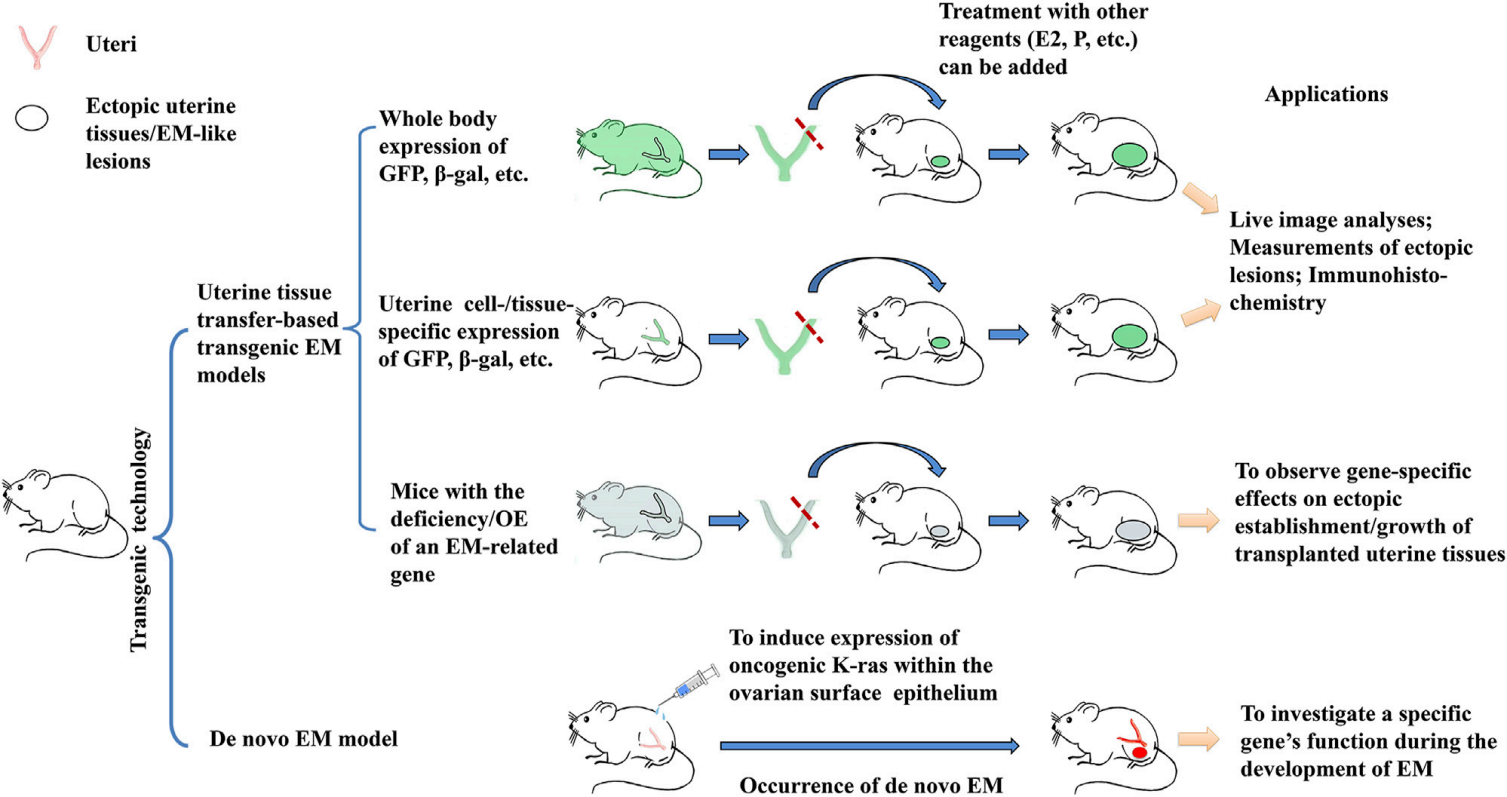
Overview of the 4 major applications of transgenic EM models. 1) Transgenic mice with whole body expression of light-emitting or chromogenic markers such as GFP and β-gal are used as sources of uterine tissues in hetero-transplantation, which enables investigators to perform live observation on the establishment/growth of ectopic uterine tissues; the reverse transplantation can be used to determine the contribution to the ectopic EM-like lesions by host tissues; 2) Transgenic mice engineered for uterine cell-/tissue-specific or pelvic tissue-specific expression of GFP or β-gal markers can serve the same purpose, but with a higher relevance to EM pathogenesis; 3) Transgenic mice with an EM-related gene knocked out (KO) or overexpressed (OE) of, when coupled to uterine tissue transfer, can be used to determine the specific gene’s *in vivo* function during the development of EM; 4) While all the 3 above applications rely on uterine tissue transfer, gene manipulation may result in the rise of ectopic endometriosis-like lesions. Such *de novo* transgenic EM models are especially helpful by offering opportunities to investigate the initiation of EM. Observation on the potential structural/functional changes in the eutopic endometrium and/or in the pelvic tissues may provide further information on EM pathogenesis.

Table 1 lists some exemplary studies and major findings with the use of transgenic EM models. As illustrated in Figure 1, transgenic mice engineered for either whole-body expression of a light-emitting or chromogenic marker are extensively applied to uterine tissue transfer-based EM models (First application in Figure 1). In comparison to conventional uterine tissue transfer models using WT mice as donor/recipient, the use of transgenic mice allows investigators to perform live image analysis or histological studies, to easily delineate the donor or recipient origin of cells in the ectopic lesions. Secondly, transgenic mice with uterine/pelvic tissue-specific expression of GFP or other markers afford the results of tissue transfer experiment a higher cell-/tissue-specificity and hence, a closer relevance to EM pathogenesis (Second application in Figure 1). Thirdly, tissue transfer from or into transgenic mice with deficiency or overexpression of an EM-related gene can be applied to characterize this gene’s *in vivo* role(s) for EM pathogenesis (Third application in Figure 1). While these tissue transfer models recapitulate the later pathogenic stages subsequent to the presence of uterine tissues in pelvic regions, it is not sufficient to elucidate questions concerning the initiation of the disease, e.g., how the uterine cell/tissues migrate to or originated in the ectopic regions. From this point of view, *de novo* EM models following gene manipulation may generate useful data in respect of a specific gene’s function for EM initiation in either uterine or pelvic tissues (Fourth application in Figure 1). The rareness of successful *de novo* EM models in literature may be partially due to the physiological differences between human and mouse, or due to the inherent requirement of a long latent time for EM to develop, which is not afforded by the murine species. Also, EM lesions are concealed inside, making them possible to escape a gross observation on transgenic mice. More research efforts focusing on the creation and characterization of *de novo* EM models are expected. Findings on the rising of *de novo* endometriotic lesions as well as structural/functional changes in eutopic uterus may fill the knowledge gaps between clinical observations and tissue transfer-based EM models. The third and fourth applications of transgenic EM models also offer superb opportunities to investigate the gene interactions with hormonal, dietetic, and environmental factors, and their potentials have not been fully exploited by EM research.

**TABLE 1:** Studies and major findings using transgenic EM models.

Gene manipulation	Title of publication	Journal, author, year	EM models	Major findings	Comments
PR KO	Intact progesterone receptors are essential to counteract the proliferative effect of estradiol in a genetically engineered mouse model of endometriosis	Fertil Steril; Fang et al. (2004)	Uterine tissues from PR OK mice were transferred to WT recipients and *vice versa*; E2 and P treatment	Intact PR in ectopic uterine tissues abolish E2-dependent or E2-independent endometriotic growth	Recipient mice were ovariectomized. PR effects in both recipient and host sides were investigated
ERα KO; ERβ KO	Role of Estrogen Receptor Signaling Required for Endometriosis-Like Lesion Establishment in a Mouse Model	Endocrinology; Burns et al., 2012	Uterine tissues from ERα or ERβ KO mice were transferred to WT recipients and *vice versa*; E2 treatment	E2 induced ectopic growth of transferred uterine tissues from WT, but not ERα knockout mice; ERα target genes were activated by E2 in ectopic lesions; E2-mediated inflammatory and angiogenic responses were predominantly through ERα	Recipient mice were ovariectomized. Only minor effects of ERβ KO were observed. Estradiol effects on EM-like tissues were largely mediated by ERα
ArKO (Cyp19 KO)	Genetic or Enzymatic Disruption of Aromatase Inhibits the Growth of Ectopic Uterine Tissue	J Clin Endocrinol Metab; Fang et al. (2002)	Auto-transplantation in ArKO and WT mice; E2 treatment	Transplants did not grow in ArKO mice; E2 treatment increased sizes of lesions in ArKO and WT mice; P450arom inhibitor reduced the sizes of lesions	It is unclear if mice were ovariectomized. Uterine horns were transferred to the bowl mesentery
Sirt1 uterine- specific OE	Role of SIRT1 and Progesterone Resistance in Normal and Abnormal Endometrium	J Clin Endocrinol Metab; Kim et al. (2022)	Auto-transplantation in mice with uterine-specific Sirt1 overexpression	SIRT1 overexpression or treatment with SIRT1 agonist increased the number of endometriotic lesions; Aberrant SIRT1 expression may confer progesterone resistance through SIRT1-PR-A protein interaction	Recipient mice were ovariectomized. SIRT1 expression was characterized in endometriotic tissues of patients as well as mouse EM models
PRNP KO/OE	PrP(C) Promotes Endometriosis Progression by Reprogramming Cholesterol Metabolism and Estrogen Biosynthesis of Endometrial Stromal Cells through PPARα Pathway	Int J Biol Sci; Peng et al. (2022)	Hetero-transplantation of uterine tissues from PRNP KO/OE to WT recipients	E2 enhanced cell survival, PrP^C^ expression, cholesterol accumulation and estrogen biosynthesis in stromal cell primary culture; Uterine tissues from PRNP KO mice produced smaller and those from PRNP OE mice produced larger, endometriotic tissues, than those from WT mice	It is unclear if mice were ovariectomized
SRC-1 KO; Mmp9 KO; TNF-αΚΟ	A new isoform of steroid receptor coactivator-1 is crucial for pathogenic progression of endometriosis	Nat Med; Han et al. (2012)	Hetero-transplantation from *SRC-1−/−*:GFP to WT; Auto-transplantation in Mmp9 and TNF-α deficient mice	Uterine tissues from *SRC-1−/−*:GFP produced smaller lesions in WT recipients; Mmp9 and TNF-α KO decreased the size of lesions; TNF-α/MMP9/SRC-1 pathway may promote EM pathogenesis	Recipient mice were ovariectomized
uterine-specific Mig-6 KO	Loss of MIG-6 results in endometrial progesterone resistance via ERBB2	Nat Commun; Yoo et al. (2022)	Transfer of WT or uterine- specific Mig-6 KO uterine tissues to WT recipients	Mig-6 deficiency increased the formation of endometriotic lesions. ERBB2 OE in endometrium with MIG-6 deficiency causes progesterone resistance	Recipient mice were ovariectomized. Downregulation of Mig-6 was confirmed in endometriotic women and baboon EM model
Leptin receptor mutant; GFP OE	Ablation of Leptin Signaling Disrupts the Establishment, Development, and Maintenance of Endometriosis-Like Lesions in a Murine Model	Endocrinology; Styer et al. (2008)	Hetero-transplantation of uterine tissues from leptin receptor mutant mice to WT recipients and *vice versa*	Leptin receptor antagonist disrupted ectopic growth of EM-like lesions; Leptin signaling is a necessary component in lesion proliferation, early vascular recruitment and angiogenesis	Recipient mice were ovariectomized. Donor mice were primed with pregnant mare serum gonadotropin or 17-β-estradiol
PPARα KO	Angiogenic and Inflammatory Alterations of Endometriotic Lesions in a Transgenic Animal Experimental Model With Loss of Expression of PPAR-Alpha Receptors	Cureus; Pergialiotis et al. (2022)	Auto-transplantation in PPARa KO and WT mice	PPARα receptors deficiency impeded the formation of endometriotic lesions as well as vascularization in the lesions	It is unclear if mice were ovariectomized. Suture was used to ensure that the mucosa would be in direct contact with the peritoneal surface
Slit2 OE	Slit2 Overexpression Results in Increased Microvessel Density and Lesion Size in Mice With Induced Endometriosis	Reprod Sci; Guo et al. (2013)	Hetero-transplantation from Slit2 OE mice to Slit2 OE or WT mice and *vice versa*	Slit2 OE increased endometriotic growth from both the donor and host sides, possibly by promoting angiogenesis	It is unclear if mice were ovariectomized. Uterine fragments were sutured to the peritoneum of lower parts of the abdomen and pelvic cavity
FKBP52 KO; Vascular epithelial cell-specific β-gal expression	Deficiency of Immunophilin FKBP52	Am J Pathol; Hirota et al. (2008)	Hetero-transplantation of uterine tissues from FKBP52 KO to WT or FKBP52 KO and *vice versa*	Fkbp52 deficiency in both donor and recipient sides promoted formation of endometriotic lesions and angiogenesis; The ectopic uterine tissues recruited blood vessel from host side	Since Fkbp52 deficient female mice had more than normal estrogenic influence due to progesterone resistance, ovariectomy is circumvented
	Promotes Endometriosis				
Diphtheria toxin receptor OE driven by CD206 promoter	CD206+ macrophage is an accelerator of endometriotic-like lesion via promoting angiogenesis in the endometriosis mouse model	Sci Rep; Ono et al. (2021)	WT endometrial fragments were injected to recipients with CD206+ macrophage depletion	The depletion of CD206+ macrophage in recipients decreased the total weight of EM-like lesions, most possibly by decreasing the proliferation of endometriotic cells and angiogenesis	It is unclear if mice were ovariectomized. While CD206 mRNA was decreased after induction with diphtheria toxin, the reduction of CD206+ cells in eutopic endometrium as well as EM-like was unconfirmed
IL-32 OE	Role of interleukin-32 in the pathogenesis of endometriosis: *in vitro*, human and transgenic mouse data	Hum Reprod; Lee et al. (2018)	Auto- transplantation in IL-32 OE and WT mice	IL-32 OE exerted pro-EM effects in both transgenic EM mouse model and endometrial cell culture	Auto-transplantation were performed in ovariectomized mice. The role of IL-32 was characterized in both Ishikawa cell culture and mouse EM model
Insertion of the *C. elegans* Fat-1 gene	Omega-3 Polyunsaturated Fatty Acids Suppress the Cystic Lesion Formation of Peritoneal Endometriosis in Transgenic Mouse Models	PLoS One; Tomio et al. (2013)	Transplantation in mice with insertion of *C. elegans* Fat-1 gene and WT mice	Uterine tissue transfer from mice with insertion of *C. elegans* Fat-1 gene to mice with the same gene manipulation produced much fewer endometriotic lesions than WT to WT transfer, and decreased levels of pro-inflammatory cytokines were found in lesions from the tissue transfer of Fat-1 insertion mice to Fat-1 insertion mice	Both donor and recipient mice were ovariectomized, and treated with estradiol
Cxcr4 KO	Loss of Cxcr4 in Endometriosis Reduces Proliferation and Lesion Number while Increasing Intraepithelial Lymphocyte Infiltration	Am J Pathol; Tal et al. (2021)	Transfer of uterine tissues with inducible Cxcr4 KO to the peritoneum of cycling host mice expressing GFP	Local CXCR4 expression is necessary for proliferation of the epithelial compartment of endometriosis lesions	Instead of ovariectomy, the estrous cycling was determined, and mice in natural cycling were used as recipients for uterine tissue transfer
*Pten* heterozygous KO in PR-expressing cells	Activated AKT Pathway Promotes Establishment of Endometriosis	Endocrinology; Kim et al. (2014)	auto-transplantation in Pten heterozygous KO mice; Treatment with AKT inhibitor	*Pten*-heterozygous mice had a diminished level of p (Ser473)-AKT in the endometrium, and produced significantly more endometriotic lesions by auto-transplantation than controls; AKT inhibitor reduced the number of endometriotic lesions	Auto-transplantation were performed in ovariectomized mice. Stromal cells from human ovarian endometrioma and endometrial stromal cells from disease-free patients and EM mouse models were all used
Oncogenic K-ras mutation	Role of K-ras and Pten in the development of mouse models of endometriosis and endometrioid ovarian cancer	Nat med; Dinulescu et al. (2005)	A *de novo* EM model was produced by expressing oncogenic K-ras within the ovarian surface epithelium	Expression of oncogenic K-ras within ovarian surface epithelium led to EM-like ovarian lesions that may arise from ovarian surface epithelium, and led to peritoneal endometriosis that may arise from uterine or tubal origin	This is the first *de novo* genetic model of peritoneal endometriosis and endometrioid ovarian adenocarcinoma
Whole body GFP OE	Luminal epithelium in endometrial fragments affects their vascularization, growth and morphological development into endometriosis-like lesions in mice	Dis Model Mech; Feng et al. (2014)	Transfer of GFP-positive uterine tissues to the dorsal skinfold chambers of GFP-negative recipients; Intravital fluorescence microscopy	The presence of uterine luminal epithelium in donor tissues negatively affected the ectopic growth of EM-like lesions in the dorsal skinfold chamber, possibly by preventing the vascular interconnection with surrounding host tissues	Instead of ovariectomy, the estrous cycling was determined. Lesions in the dorsal skinfold chambers are easily accessible for intravital microscopic analyses

Note: KO, knockout; OE, overexpression.

Comparing to *in vitro* studies using cell culture, the *in vivo* models are more advantageous to investigate the cell/tissue interactions in a more disease-relevant manner, whereas the *in vitro* systems may be more practical and efficient for analyzing molecular mechanisms/pathways. It appears that animal models involve more variables than cell cultures, which renders the data interpretation of the former more uncertainty than the latter. For example, during creation of EM models, different research groups applied endometrial tissues that are minced to different sizes, and the tissue pieces could be injected into the pelvic cavity or sutured in pelvic cavity. Moreover, in spite of the well-recognized, dominant role of estrogen for EM development, some EM model studies did not perform ovariectomy to control the estrogen level or used mice from the same estrus cycle (Table 1), raising a quality control issue. All these technical divergences may lead to significant variations in the tissue interactions and growth of EM-like tissues. Since the biological implications of these technical variables are not precisely defined, comparison and interpretation of data from EM animal models could be a challenging task.

It should be mentioned that most transgenic EM models used so far are in static gene deficiency/overexpression status since animals’ day one of lives, from the stage of fertilized egg. Inducible transgenic models that set a gene’s deficiency/overexpression in a timely controlled manner would be more effective to characterize EM pathogenesis in a temporal relevance. The creation of transgenic EM models might be limited by the fatal phenotype that is often inherent to the knockout of a critical gene. Due to technical hurdles the epigenetic components of EM pathogenesis are difficult to be recapitulated by the transgenic EM models. In addition, due to the species differences and artificial nature of tissue transfer, necessary prudence should be exercised during data interpretation as well as when extrapolating data from mouse models to human EM. Nevertheless, transgenic mice have provided a powerful *in vivo* tool for EM studies, and their applications have much advanced our understanding of EM pathogenesis concerning hormonal regulation, angiogenesis and inflammation, especially when coupled to advanced live imaging techniques and multi-omics analyses.
