# Supplementary material for: Transgenic mice applications in the study of endometriosis pathogenesis
Source: Front Cell Dev Biol. 2024 Jun 12;12:1376414. doi: 10.3389/fcell.2024.1376414 (PMC11199864; doi:10.3389/fcell.2024.1376414)
Supplement: Supplementary file 1 [file Table1.DOCX]

**Table 1.** Studies and major findings using transgenic EM models

| **Gene Manipulation** | **Title of Publication** | **Journal, Author, Year** | **EM Models** | **Major Findings** | **Comments** |
| --- | --- | --- | --- | --- | --- |
| PR KO | Intact progesterone receptors are essential to counteract the proliferative effect of estradiol in a genetically engineered mouse model of endometriosis | Fertil Steril; Fang et al; 2004 | Uterine tissues from PR OK mice were transferred to WT recipients and vice versa; E2 and P treatment | Intact PR in ectopic uterine tissues abolish E2-dependent or E2-independent endometriotic growth | Recipient mice were ovariectomized. PR effects in both recipient and host sides were investigated |
|  |  |  |  |  |  |
| ERα KO; ERβ KO | Role of Estrogen Receptor Signaling Required for Endometriosis-Like Lesion Establishment in a Mouse Model | Endocrinology; Burns et al; 2012 | Uterine tissues from ERα or ERβ KO mice were transferred to WT recipients and vice versa; E2 treatment | E2 induced ectopic growth of transferred uterine tissues from WT, but not ERα knockout mice; ERα target genes were activated by E2 in ectopic lesions; E2-mediated inflammatory and angiogenic responses were predominantly through ERα | Recipient mice were ovariectomized. Only minor effects of ERβ KO were observed. Estradiol effects on EM-like tissues were largely mediated by ERα |
|  |  |  |  |  |  |
| ArKO (Cyp19 KO) | Genetic or Enzymatic Disruption of Aromatase Inhibits the Growth of Ectopic Uterine Tissue | J Clin Endocrinol Metab; Fang et al; 2002 | Auto-transplantation in ArKO and WT mice; E2 treatment | Transplants did not grow in ArKO mice; E2 treatment increased size of lesions in ArKO and WT mice; P450arom inhibitor reduced the size of lesions | It is unclear if mice were ovariectomized. Uterine horns were transferred to the bowl mesentery |
|  |  |  |  |  |  |
| Sirt1 uterine- specific OE | Role of SIRT1 and Progesterone Resistance in Normal and Abnormal Endometrium | J Clin Endocrinol Metab; Kim et al; 2022 | Auto-transplantation in mice with uterine-specific Sirt1 overexpression | SIRT1 overexpression or treatment with SIRT1 agonist increased the number of endometriotic lesions; Aberrant SIRT1 expression may confer progesterone resistance through SIRT1-PR-A protein interaction | Recipient mice were ovariectomized. SIRT1 expression was characterized in endometriotic tissues of patients as well as mouse EM models. |
| PRNP KO /OE | PrP(C) Promotes Endometriosis Progression by Reprogramming Cholesterol Metabolism and Estrogen Biosynthesis of Endometrial Stromal Cells through PPARα Pathway | Int J Biol Sci; Peng et al; 2022 | Hetero-transplantation of uterine tissues from PRNP KO /OE to WT recipients | E2 enhanced cell survival, PrP^C^ expression, cholesterol accumulation and estrogen biosynthesis in stromal cell primary culture; Uterine tissues from PRNP KO mice produced smaller and those from PRNP OE mice produced larger, endometriotic tissues, than those from WT mice | It is unclear if mice were ovariectomized |
|  |  |  |  |  |  |
| SRC-1 KO; Mmp9 KO; TNF-  αKO | A new isoform of steroid receptor coactivator-1 is crucial for pathogenic progression of endometriosis | Nat Med; Han, S.J., et al; 2012 | Hetero-transplantation from *SRC-1-/-*:GFP to WT; Auto-transplantation in Mmp9 and TNF-α deficient mice | Uterine tissues from *SRC-1-/-*:GFP produced smaller lesions in WT recipients; Mmp9 and TNF-α KO decreased the size of lesions; TNF-α/MMP9/SRC-1 pathway may promote EM pathogenesis | Recipient mice were ovariectomized |
|  |  |  |  |  |  |
| uterine-specific Mig-6 KO | Loss of MIG-6 results in endometrial progesterone resistance via ERBB2 | Nat Commun; Yoo et al; 2022 | Transfer WT or uterine- specific Mig-6 KO uterine tissues to WT recipients | Mig-6 deficiency increased the formation of endometriotic lesions. ERBB2 OE in endometrium with MIG-6 deficiency causes progesterone resistance | Recipient mice were ovariectomized. Down regulation of MIG-6 was confirmed in endometriotic women and baboon EM model |
|  |  |  |  |  |  |
| Leptin receptor mutant; GFP OE | Ablation of Leptin Signaling Disrupts the Establishment, Development, and Maintenance of Endometriosis-Like Lesions in a Murine Model | Endocrinology; Styer et al; 2008 | Hetero-transplantation of uterine tissues from leptin receptor mutant mice to WT recipient and vice versa | Leptin receptor antagonist disrupted ectopic growth of EM-like lesions; Leptin signaling is a necessary component in lesion proliferation, early vascular recruitment and angiogenesis | Recipient mice were ovariectomized. Donor mice were primed with pregnant mare serum gonadotropin or 17-β-estradiol |
|  |  |  |  |  |  |
| PPARα KO | Angiogenic and Inflammatory Alterations of Endometriotic Lesions in a Transgenic Animal Experimental Model With Loss of Expression of PPAR-Alpha Receptors | Cureus; Pergialiotis et al; 2022 | auto-transplantation in PPARa KO and WT mice | PPARα deficiency impeded the formation of endometriotic lesions as well as vascularization in the lesions | It is unclear if mice were ovariectomized. Suture was used to ensure that the mucosa would be in direct contact with the peritoneal surface |
| Slit2 OE | Slit2 Overexpression Results in Increased Microvessel Density and Lesion Size in Mice With Induced Endometriosis | Reprod Sci; Guo et al; 2013 | Hetero-transplantation from Slit2 OE mice to Slit2 OE or WT mice and vice versa | Slit2 OE increased endometriotic growth from both the donor and host sides, possibly by promoting angiogenesis | It is unclear if mice were ovariectomized. Uterine fragments were sutured to the peritoneum of lower parts of the abdomen and pelvic cavity |
|  |  |  |  |  |  |
| FKBP52 KO; Vascular epithelial cell-specific β-gal expression | Deficiency of Immunophilin FKBP52  Promotes Endometriosis | Am J Pathol ; Hirota et al; 2008 | Hetero-transplantation of uterine tissues from FKBP52 KO to WT or FKBP52 KO and vice versa | Fkbp52 deficiency in both donor and recipient sides promoted formation of endometriotic lesions and angiogenesis; The ectopic uterine tissues recruited blood vessel from host side | Since Fkbp52 deficient female mice had more than normal estrogenic influence due to progesterone resistance, ovariectomy is circumvented |
|  |  |  |  |  |  |
| Diphtheria toxin receptor OE driven by CD206 promoter | CD206+ macrophage is an accelerator of endometriotic-like lesion via promoting angiogenesis in the endometriosis mouse model | Sci Rep; Ono et al; 2021 | WT endometrial fragments were injected to recipients with CD206+ macrophage depletion | The depletion of CD206+ macrophage decreased the total weight of EM-like lesions, most possibly by decreasing the proliferation of endometriotic cells and angiogenesis | It is unclear if mice were ovariectomized. While CD206 mRNA was decreased after induction with diphtheria toxin, the reduction of CD206+ cells in eutopic endometrium as well as EM-like was unconfirmed |
| IL-32 OE | Role of interleukin-32 in the pathogenesis of endometriosis: in vitro, human and transgenic mouse data | Hum Reprod; Lee et al; 2018 | Auto- transplantation in IL-32 OE and WT mice | IL-32 OE exerted pro-EM effects in both transgenic EM mouse model and endometrial cell culture | Auto-transplantation were performed in ovariectomized mice. The role of IL-32 was characterized in both Ishikawa cell culture and mouse EM model |
|  |  |  |  |  |  |
| Insertion of the *C. elegans* Fat-1 gene | Omega-3 Polyunsaturated Fatty Acids Suppress the Cystic Lesion Formation of Peritoneal Endometriosis in Transgenic Mouse Models | PLoS One; Tomio et al; 2013 | Transplantation in mice with insertion of *C. elegans* Fat-1 gene and WT mice | Uterine tissue transfer from mice with insertion of *C. elegans* Fat-1 gene to mice with the same gene manipulation produced much fewer endometriotic lesions than WT to WT transfer, and decreased levels of pro-inflammatory cytokines were found in lesions from the tissue transfer of Fat-1 insertion mice to Fat-1 insertion mice | Both donor and recipient mice were ovariectomized, and treated with estradiol |
|  |  |  |  |  |  |
| Cxcr4 KO | Loss of Cxcr4 in Endometriosis Reduces Proliferation and Lesion Number while Increasing Intraepithelial Lymphocyte Infiltration | Am J Pathol; Tal et al; 2021 | Transfer of uterine tissues with inducible Cxcr4 KO to the peritoneum of cycling host mice expressing GFP | Local CXCR4 expression is necessary for proliferation of the epithelial compartment of endometriosis lesions | Instead of ovariectomy, the estrous cycling was determined, and mice in natural cycling were used as recipients for uterine tissue transfer |
|  |  |  |  |  |  |
| *Pten* heterozygous KO in PR-expressing cells | Activated AKT Pathway Promotes Establishment of  Endometriosis | Endocrinology; Kim et al; 2014 | auto-transplantation in Pten heterozygous KO mice; Treatment with AKT inhibitor | *Pten*-heterozygous mice had a diminished level of p(Ser473)-AKT in the endometrium, and produced significantly more endometriotic lesions than controls; AKT inhibitor reduced the number of endometriotic lesions | Auto-transplantation were performed in ovariectomized mice. Stromal cells from human ovarian endometrioma and endometrial stromal cells from disease-free patients and EM mouse models were all used |
|  |  |  |  |  |  |
| Oncogenic K-ras mutation | Role of K-ras and Pten in the development of mouse models of endometriosis and endometrioid ovarian cancer | Nat med; Dinulescu et al; 2005 | A de novo EM model was produced by expressing oncogenic K-ras within the ovarian surface epithelium | Expression of oncogenic K-ras within ovarian surface epithelium led to EM-like lesions on ovarian surface that may arise from ovarian surface epithelium, and led to peritoneal endometriosis that may arise from uterine or tubal origin | This is the first de novo genetic model of peritoneal endometriosis and endometrioid ovarian adenocarcinoma |
|  |  |  |  |  |  |
| Whole body GFP OE | Luminal epithelium in endometrial fragments affects their vascularization, growth and morphological development into endometriosis-like lesions in mice | Dis Model Mech; Feng et al; 2014 | Transfer of GFP-positive uterine tissues to the dorsal skinfold chambers of GFP-negative recipients; Intravital fluorescence microscopy | The presence of uterine luminal epithelium in donor tissues negatively affected the ectopic growth of EM-like lesions in the dorsal skinfold chamber, possibly by preventing the vascular interconnection with surrounding host tissues | Instead of ovariectomy, the estrous cycling was determined. Lesions in the dorsal skinfold chambers are easily accessible for intravital microscopic analyses |

Note: KO, knockout; OE, overexpression
